# Coherence, transformation, and breakdown in living systems: part I — the common higher-order regulatory architecture (CHORA) hypothesis

**DOI:** 10.3389/fsysb.2026.1910966

**Published:** 2026-10-02

**Authors:** Andreas Nicolaides

**Affiliations:** Independent Researcher, Harrogate, United Kingdom

**Keywords:** CHORA hypothesis, entrenchment, evolutionary coherence, germline regulatory systems, higher-order regulatory architecture, systemic disease, systems biology, transposable elements

## Abstract

The coherence of living systems extends beyond the lifespan of the individual organism: it persists across biological lineages, through the turnover of organisms and change across deep evolutionary time. This paper terms this phenomenon evolutionary coherence, the lineage-scale expression of a more general property, biological coherence, whose organism-scale expression systems biology has characterised while its evolutionary counterpart has not. Biological coherence is analysed through four aspects — stability, transformation, connectivity, and coordination — each expressed at both the lineage and the organism scale. Their lineage-level manifestations appear in familiar but unreconciled patterns: persistent morphologies, punctuated transformation, structured hybridisation, and the channelled forms evolutionary change repeatedly takes. At the organism scale the same aspects govern the maintenance of organised function and its breakdown, including the convergence of distinct systemic diseases on shared signatures of regulatory dysfunction. Such patterns are not readily accommodated by the foundational ideas of evolutionary biology and biomedicine, which engage auxiliary hypotheses to account for them; some point toward higher-order organisation but stop short of articulating it as primary. Proceeding by abductive inference, the paper specifies the requirements a unifying account must meet, then surveys the molecular evidence and develops a systems model of their operation. The architectures examined are hierarchical gene regulatory networks, bow-tie physiological regulation, germline regulatory systems, the regulation of repetitive DNA, and centromere identity. The framework is articulated as the CHORA Hypothesis: that a Common Higher-Order Regulatory Architecture, common to the organism and the lineage, operates as an integrated level across organisms and generations; that the germline is its principal conduit; that the features sustaining coherence also underlie its failure; and that its disruption at the organism level underlies these convergent signatures of dysfunction. The architecture is not a further agency but the largely conserved higher-order organisation realised by established molecular processes — transposable-element activity foremost among them — and through which coherence is sustained. These processes are its molecular signatures; the architecture’s own nature is left open. Health and entrenched disease are, on this account, alternative regimes of the one architecture. Part II develops the discriminating predictions and systemic-health implications.

## 1 Introduction

Living systems maintain coherent organisation, regulate their internal dynamics, and either sustain that coherence over time or fail to do so. These capacities determine biological viability: whether an organism remains healthy or succumbs to disease, and whether a lineage persists or breaks down. How such coherence is sustained has been extensively engaged at the level of the individual organism, where systems biology has characterised the regulatory architectures through which developmental and physiological order is maintained across the lifespan. Less developed is the corresponding question at the level of the lineage: how is organised biological function sustained across deep time, through the continual turnover of organisms, the variation of conditions, and the accumulation of change? This is the phenomenon the present paper terms evolutionary coherence.

Evolutionary coherence is not a phenomenon evolutionary biology has consolidated under a single name. The standard framework theorises evolutionary change in terms of variation, inheritance, and selection (Futuyma and Kirkpatrick, 2022), with the persistence of organised function across deep time treated as the cumulative outcome of those random processes acting on phenotypic variation. When examined directly, lineage-level coherence exhibits patterns unexpected under the standard framework alone: persistence over deep time with directional change rare and stasis common across many lineages (Simpson, 1944; Hunt, 2007), and the recurrence of organisational features across taxa separated by enormous evolutionary distances.

These patterns become intelligible when their architectural substrates are examined. Homologous structures share homologous regulatory architectures: the bilaterian body plan is sustained across the animal kingdom by deeply embedded gene regulatory network kernels conserved across hundreds of millions of years (Davidson and Erwin, 2006), with comparable conservation in bow-tie physiological regulation, germline regulatory systems, the surveillance architectures channelling repetitive DNA, and centromere identity. These substrates, examined in Section 5, are what allow a lineage to produce coherent organisms across deep time.

The same substrates, disrupted at the level of the individual organism, are observed in systemic disease, with conditions whose proximate causes differ substantially (cancers, autoimmune conditions, neurodegenerative diseases, age-related decline) converging on shared signatures of regulatory dysfunction (Hanahan and Weinberg, 2011; López-Otín et al., 2013; Hanahan, 2022). The convergence is intelligible through the evolutionary depth of the architectures whose disruption produces the pathologies and is developed in Section 5.

The anomalous patterns surveyed across these areas point in the same direction. The standard frameworks of evolutionary biology and biomedicine recover them through domain-specific hypotheses, while broader organisational traditions (Waddington, 1957; Moreno and Mossio, 2015; Laland et al., 2015) supplement those frameworks without displacing their core explanatory commitments. There is a pressing need for system-level alternatives to the gene-centred view, a notable example being Genome Architecture Theory, advanced by Heng and Heng (2022), Heng and Heng (2023). The present paper adopts a similar system-level approach, though differing in important respects (see the Discussion). It develops the suggestion that the observed patterns are intelligible as the evolutionary behaviour of a single higher-order regulatory architecture sustaining coherence over deep time. The argument proceeds by abductive inference (Peirce, 1931; Lipton, 2004), asking what form of organisation would render the recurrent patterns intelligible.

The proposed framework is articulated in Section 7 as the CHORA Hypothesis: that living systems possess a Common Higher-Order Regulatory Architecture whose integrity sustains biological coherence across time, whose operation permits ordered transformation, whose disruption underlies characteristic modes of breakdown, and which is propagated across generations principally through the germline.

A note on terminology. Systemic disease is used here in an architectural rather than a clinical sense, the clinical sense denoting disease involving several organ systems. Here it denotes disorder of regulation where the architecture sustaining within-organism coherence has been disrupted, whether or not more than one organ system is clinically involved. The category is the counterpart of systemic health, the two being alternative regimes of one architecture rather than opposing states. Where the regime has become self-sustaining, the term entrenched systemic disease is used, which is the subset this framework claims; not all systemic disease is of that kind.

The evidence considered is primarily eukaryotic and especially metazoan, where the molecular architectures are best characterised and the health-relevant phenomena chiefly concern multicellular organisms. This focus reflects the paper’s link between regulatory architecture and systemic disease, not a claim that CHORA-like architectures are confined to eukaryotes. Evidence for analogous architectures in bacteria and archaea is substantial but reserved for systematic treatment elsewhere.

## 2 Evolutionary coherence

The phenomenon at the centre of this paper is evolutionary coherence: the persistence of organised biological function across deep time, sustained by architectures whose own continuity is what makes lineage continuity possible. A fish, a sea urchin, and the chordate ancestor of both share recognisable patterns of body-plan organisation; the regulatory architecture that produced them in the ancestor and produces them in their descendants is essentially the same, conserved through hundreds of millions of generations. Evolutionary coherence names the persistence of this architecture across evolutionary time, its capacity to produce coherent, viable organisms generation after generation despite the continual turnover of individuals, environmental variation, and the accumulation of genetic and epigenetic change.

The cross-scale parallels between lineage-level and organism-level expressions are neither heuristic analogies nor technical homologies: the two scales are related not by descent from a common ancestral character, but by shared organisational principles realised through different proximate mechanisms, unlike the standard cross-taxa homology of structures such as the forelimb or the conserved regulatory architecture of eye development. Sections 3, 5, 6 develop the case for their common organisational basis.

Evolutionary coherence must be distinguished from within-organism coherence, and from a narrower use of the term for the correlated evolution of the components of a brain structure (Barton et al., 2003); the present usage is broader, denoting the persistence of organised function across deep time. Within-organism coherence maintains organised function across an individual lifespan; evolutionary coherence does so across a lineage’s succession of organisms. These are distinct but connected scales of biological coherence, each generation’s within-organism coherence depending on the architecture inherited from previous ones. This paper principally concerns the lineage scale.

The persistence of evolutionary coherence is not a guarantee of biological viability. Lineages do go extinct, and the conditions that maintain evolutionary coherence are not invulnerable. Where lineages persist across deep evolutionary intervals, they do so through regulatory architectures reproduced across generations with sufficient fidelity to maintain characteristic organisation. The persistence is not the absence of change (lineages do change, sometimes substantially) but the maintenance of a coherent organisational identity across change.

### 2.1 The four aspects of biological coherence

Biological coherence is not a single property but a phenomenon with four distinguishable aspects, each expressed at the level of the lineage, where it constitutes evolutionary coherence, and of the individual organism, where it constitutes within-organism coherence. Two of them, stability and transformation, are most directly relevant to how organised function is sustained and how it breaks down, the central concerns of the Research Topic in which this paper appears, and are developed in their own subsections below. The other two are noted here and reserved for separate work. Connectivity is the maintenance of regulated boundaries with controlled exchange across them, expressed at the lineage level as structured hybridisation and introgression and at the organism level as tissue-compartment integrity and immune self-recognition. Coordination is the joint operation of multiple regulatory processes defining the system’s functional state, expressed at the lineage level as the channelled generation of variation and at the organism level as the coordinated activity of signalling pathways, regulatory networks, and cell populations.

Connectivity bears most directly on lineage boundaries and speciation, where the framework’s claims concerning regulated exchange between architecturally distinct systems have particular force; coordination bears on the structure of evolutionary trajectories and the convergent outcomes that follow from architectural channelling. Both are developed in a companion paper on speciation.

### 2.2 Stability

Stability, considered as an aspect of biological coherence, is the persistence of architectural organisation through time: across the lifespan at the level of the organism, and across long evolutionary intervals at the level of the lineage. At the phylogenetic level, its clearest manifestation is the persistence of characteristic lineage organisation across such intervals; the pattern was articulated in general terms by Simpson (1944) and was subsequently the focus of the punctuated equilibria proposal (Eldredge and Gould, 1972; Gould, 2002). How often stasis predominates over gradual change remains debated, but pronounced intervals of stasis are broadly documented as common across many clades, with directional change rare (Hunt, 2007). What is striking is not merely the absence of change but the active maintenance of characteristic organisation despite continual perturbation.

A second phylogenetic-level manifestation of stability is observed in the persistence of organisational features across taxa. Homologous structures, recognisable across lineages separated by enormous evolutionary distances, exhibit shared organisational principles whose conservation is striking even when the morphological forms in which they are realised differ substantially. The vertebrate forelimb across mammals, birds, reptiles, and amphibians; the conserved Hox gene clusters across bilaterians; the deeply homologous regulatory architectures underlying eye development across animals despite the enormous diversity of eye structures (Shubin, Tabin, and Carroll, 2009): all are cases where the same underlying organisation appears across taxa whose common ancestors lived hundreds of millions of years ago.

These phylogenetic-level manifestations are sustained by conservation at the level of regulatory architecture, examined in Section 5; this conservation is what Shubin, Tabin, and Carroll term deep homology, in which homologous structures share homologous regulatory architectures.

The breakdown of stability is observed at the organism level in age-related decline, the loss of tissue identity in oncogenesis, and the erosion of homeostatic capacity in chronic conditions; and at the phylogenetic level in the hybrid incompatibility that follows divergence of centromeric architecture and in transgenerational erosion of germline integrity: expressions, at their respective scales, of disruption to the same architectural integrity whose maintenance sustains both coherent organisms and coherent lineages over deep time.

### 2.3 Transformation

Transformation, considered as an aspect of biological coherence, is the capacity of organised systems to undergo structural change without loss of biological viability: change that preserves coherence at a higher level even as it modifies organisation at a more particular one. At the phylogenetic level, transformation is observed as the structured reorganisations through which lineages undergo substantial change: transitions often relatively rapid in geological terms, clustering around particular periods or types of reorganisation, and frequently associated with shifts in developmental regulatory organisation rather than the gradual accumulation of small modifications (Eldredge and Gould, 1972; Davidson and Erwin, 2006; Erwin, 2007). What is striking is not merely that lineages change but that the changes are structured, occurring preferentially at particular architectural levels.

This is structured transformation in a strong sense: the architectures that sustain evolutionary coherence do not preclude change but channel it, with modification possible at some levels and catastrophic at others (Davidson and Erwin, 2006; Erwin and Davidson, 2009). The transformation is regulated by established molecular processes. Transposable elements figure in two of the five architectures presented in Section 5, the germline regulatory systems and the regulation of repetitive DNA; their causal role in transformation is part of a much wider set of molecular processes, and is fully developed in the companion paper on speciation. The same logic operates at the physiological and germline levels, where regulated reprogramming is itself transformation at the cross-generational scale, transmitting not a fixed inventory of regulatory states but the architecture through which each generation’s regulatory configuration is re-established (Reik and Walter, 2001; Hackett and Surani, 2013).

The breakdown of transformation is observed at the organism level as maladaptive reorganisation: oncogenic transformation, autoimmune transitions, and the entrenchment of dysregulated metabolic and inflammatory states (Medzhitov, 2008; Hanahan and Weinberg, 2011; Holohan et al., 2013). In each, architectural circuits that under other conditions sustain healthy reorganisation come, once sufficiently displaced, to sustain alternative regimes from which return is difficult. Breakdown of transformation at the lineage level cannot be illustrated in the same way: a lineage whose ordered transformation broke down catastrophically would leave no viable descendant to record: the ordinary survivorship condition of evolutionary evidence, not a limitation peculiar to this account.

## 3 The explanatory gap: how the standard frameworks engage biological coherence

The patterns described in Section 2 raise a question neither the standard evolutionary framework nor contemporary biomedicine addresses directly. Evolutionary biology theorises variation and selection at the population level, but not the maintenance of evolutionary coherence across the lineage over deep time; biomedicine addresses organismal function, but not within-organism coherence and its breakdown in systemic disease. Both point towards a common architectural level neither framework treats as primary.

### 3.1 Evolutionary coherence and the standard evolutionary framework

The standard framework of evolutionary biology provides a well-established account of how populations change over time. Variation arises through mutation, recombination, and related processes; inheritance transmits variants across generations; and selection, together with drift, alters the frequency of variants in ways that produce adaptation and diversification (Futuyma and Kirkpatrick, 2022). For phenomena that fall within its proper scope (allele frequency change, microevolutionary adaptation, the population-level dynamics of well-defined traits), the framework is theoretically deep, empirically tractable, and explanatorily successful.

Its predictions fit less well, however, for evolutionary coherence specifically. While gradualism predicts cumulative directional change as the typical mode of lineage evolution, the patterns observed differ. Lineages persist with characteristic organisation across long evolutionary intervals (Simpson, 1944; Hunt, 2007; Erwin, 2007); regulatory architectures are conserved across phylogenetic distances despite enormous diversity in selective regimes (Csete and Doyle, 2004; Davidson and Erwin, 2006); body-plan transitions are structured rather than continuous, concentrated at intermediate levels of regulatory hierarchy (Erwin and Davidson, 2009). These patterns sit awkwardly with what the gradualist account, on its own, would lead one to expect.

Where the framework’s predictions do not match observation, auxiliary hypotheses have been introduced to accommodate the discrepancy, in the pattern Lakatos (1970) called the protective belt with which a research programme shields its core from anomaly, an analysis applied to evolutionary theory by Pievani (2012). Punctuated equilibria (Eldredge and Gould, 1972; Gould, 2002) was the most prominent of these auxiliaries, proposing that the temporal pattern of stasis-and-transition observed in the fossil record requires an account beyond cumulative selection acting on phenotypic variation. Canalisation, evolvability, and developmental constraint emerged from the developmental biology literature to address the structured availability of variation: that lineages cannot vary in arbitrary directions, that some forms of variation are catastrophic while others are tolerated, and that the trajectories accessible to a lineage are shaped by the regulatory architecture it already possesses (Wagner, 2005; Pigliucci and Müller, 2010). For the conservation of physiological architectures (the bow-tie organisation of metabolism, the integrated stress response, circadian regulation), the response has typically been to invoke generic stabilising selection on essential function, but its sufficiency as a complete account of character conservation has been questioned within quantitative genetics (Hansen and Houle, 2004); the appeal does not by itself explain why the same architectural organisation, rather than any functionally equivalent alternative, recurs across lineages whose selective regimes differ substantially. Conservation of function does not entail conservation of architecture.

The Extended Evolutionary Synthesis (Pigliucci and Müller, 2010; Laland et al., 2015; Müller, 2017) consolidates and extends these earlier developments into the most ambitious contemporary attempt to broaden the standard framework. It identifies developmental processes as structuring the generation of variation, niche construction as modifying selective environments, and inclusive inheritance as operating through multiple channels beyond the genome alone. The concepts it draws together, canalisation, developmental constraint, evolvability, and structured variation, are now substantially part of contemporary evolutionary biology, and the phenomena they describe are not in dispute. What is in question is their explanatory status: whether these phenomena are best understood, as the standard framework treats them, as separate factors supplementing selection, or as expressions of a single underlying architecture. Each was introduced to accommodate a pattern the selection-centred framework did not predict, and within that framework each remains supplementary to it rather than a primary explanatory level in its own right.

A selectionist counterargument holds that the recurrent architectural pattern is itself a product of selection: such architectures are selectively favoured solutions to the general problem of building complex systems that are both robust and flexible, and recur across regulatory domains because selection independently favours them (Csete and Doyle, 2002; Wagner, 2005; Friedlander et al., 2015). The architectural account does not deny that selection may have acted on these architectures. What it does deny is that the persistent recurrence of the architectural pattern is fully explained by selection acting on phenotypic outputs. The difference is one of unifying power: a single architectural account renders the recurrent pattern intelligible across regulatory domains, whereas an account resting on selection acting on phenotypic outputs addresses each instance of the pattern separately. The specific difficulties that the gradient-selection account encounters in producing architectures with the features observed — catastrophic failure at central nodes, conservation tracking architectural centrality, propagation by mechanisms not encoded in the sequence selection acts on — are developed in Section 5.

### 3.2 Within-organism coherence and the standard biomedical framework

Contemporary biomedicine exhibits a structurally similar pattern in the breakdown of within-organism coherence in systemic disease. The component-level framework, which has dominated biomedical thinking for decades, treats disease as the malfunction of distinct molecular components and pathways. Where pathology localises to a specific lesion (a mutation, a disrupted pathway, a deficient signalling molecule), this framework has enabled interventions of considerable precision, from targeted cancer therapies to monoclonal antibodies and gene therapies for defined disorders (Alberts et al., 2022; Weinberg, 2023).

The framework’s predictions begin to fail when applied to the breakdown of within-organism coherence as a phenomenon. Systemic diseases whose proximate causes differ substantially (cancers, autoimmune conditions, neurodegenerative diseases, age-related decline) converge on shared signatures of regulatory dysfunction (Hanahan and Weinberg, 2011; López-Otín et al., 2013; Hanahan, 2022). The component-level framework does not readily anticipate this convergence: on its own it would lead one to expect pathologies with different proximate causes to exhibit different signatures. The convergence becomes intelligible only when the architectures whose disruption produces the pathologies are themselves engaged as the explanatory level: the same architectures whose conservation, at the lineage scale, sustains evolutionary coherence.

Network medicine (Barabási et al., 2011; Menche et al., 2015) and the hallmarks frameworks (Hanahan and Weinberg, 2011; López-Otín et al., 2013; Hanahan, 2022) have been introduced as auxiliary hypotheses to the component-level framework, each engaging the regulatory and architectural dimensions of pathology that component-level analysis leaves underexplored. Network medicine treats disease as the perturbation of network organisation rather than the failure of individual components, and identifies disease modules whose overlap across distinct conditions points toward shared architectural substrates. The hallmarks frameworks document a set of signatures — genomic instability, deregulated nutrient sensing, cellular senescence, altered intercellular communication — that recur across cancer, ageing, immune dysfunction, and neurodegeneration, pathologies whose proximate mechanisms differ substantially. Both engage what the component-level framework leaves aside, and both point toward regulatory architecture as the level at which the convergence and recurrence are intelligible; yet both stop at describing these regularities rather than making regulatory architecture the primary explanatory level from which they follow — the step the present framework takes.

### 3.3 The shape of the shared gap

The two frameworks, considered together, exhibit a structurally parallel limitation. Both have introduced auxiliary hypotheses where their predictions do not match observation. These differ in their specifics but share an underlying character: each engages a feature of regulatory organisation that the framework employing it does not theorise as a primary explanatory level, and each points toward the architectural level the present paper articulates. That these hypotheses are now well established does not alter their logical role: an auxiliary hypothesis is one introduced to accommodate what the core account did not predict, and it remains auxiliary in that sense however widely it is accepted. The relevant contrast is one of parsimony. A single architectural principle from which these patterns follow requires fewer independent assumptions than a set of separately introduced hypotheses, each accommodating one pattern after the fact, and it yields the patterns as predictions rather than accommodations. A well-established synthesis of many such hypotheses is not, on that account, preferable to a single principle of comparable adequacy; it is less parsimonious.

The shared gap is therefore one of articulation, not engagement: the present paper develops the suggestion that this architectural level admits of substantive characterisation as a primary explanatory level. The remainder of the paper develops this suggestion: Section 4 sets out what an account of biological coherence at this level must address, and Sections 5–7 develop the molecular evidence, the systems model, and the hypothesis that emerges.

## 4 Requirements for an account of biological coherence

Addressing these patterns directly requires meeting requirements at both the lineage and organism scales, treating evolutionary and within-organism coherence as expressions of a single architecture. The requirements below specify what their joint satisfaction by one architecture would demand.

Five appear to follow, not offered as an exhaustive list.

### 4.1 Maintenance of architectural organisation across deep time

The first requirement is that the account address the maintenance of evolutionary coherence: the persistence of organised biological function across long evolutionary intervals despite the continual turnover of organisms, the variation of environments, and the accumulation of genetic and epigenetic change. Lineages persist with characteristic organisation across deep time not because the organisms within them are static (they are not), but because the architectures producing those organisms are sustained across the succession of generations. The account must therefore address what sustains this persistence and what conditions are required for the architecture to be reproduced with sufficient fidelity to maintain the lineage’s organisational identity.

### 4.2 Ordered transformation

The account must also address the capacity of these architectures to accommodate ordered change without loss of coherence. They are not static. Lineages undergo substantial transformation: in the regulatory architectures expressed during development, in the physiological reorganisations of the life course, in the regulated reprogramming of the germline, and at the lineage level itself in the structured reorganisations that punctuate evolutionary stasis. What distinguishes ordered transformation from disintegrative change is that it occurs within the architecture rather than disrupting it, with coherence preserved at a higher level even as organisation is modified at a more particular one. The account must therefore address how a lineage’s available trajectories are shaped by the architecture it already possesses, how variation is channelled through structured rather than arbitrary forms, and how the architecture itself can be reconfigured while the lineage’s continuity is preserved.

### 4.3 Threshold-dependent transition

A third requirement concerns the limits of the envelope within which biological coherence and ordered transformation are sustained, and what occurs when those limits are exceeded. The architectures do not exhibit continuous gradations of function across all conditions: they display characteristic regimes separated by thresholds at which qualitative transitions occur. The account must therefore address what produces this threshold-dependent character, and what determines the threshold’s location for a given architecture.

### 4.4 Self-sustaining dysregulation

Closely connected to threshold-dependent transition is the entrenchment of dysregulated states. Not all transitions out of the operating envelope are self-limiting: in some cases the architectural circuits that ordinarily return the system toward its envelope come, when displaced sufficiently, to stabilise a dysregulated regime rather than correct it. This requirement is demanding, asking the account to treat the stability of normal function and of certain abnormal states as expressions of the same architectural principles. The account must address why some breakdowns are self-correcting while others become self-sustaining, a question bearing directly on within-organism coherence.

### 4.5 Propagation across generations

A final requirement concerns propagation. Evolutionary coherence is the persistence of organised biological function across the succession of organisms that a lineage produces, and the account must therefore address how the architecture that sustains coherence within any given generation is transmitted such that descendant systems inherit the conditions for evolutionary coherence to be re-established. The germline is the principal site at which this requirement is met, because it is there that the architecture governing what is transmitted (epigenetic reprogramming machinery, chromosomal organisation, and the regulatory constraints on transposable elements and repetitive DNA) is sustained across generations. What is propagated is not a fixed inventory of regulatory states but the architecture and the conditions under which each generation’s regulatory organisation can be reconstructed. The account must therefore address what is transmitted, how, and what failures of transmission imply for the lineage.

### 4.6 The requirements considered together

The five requirements are not independent: maintenance defines the operating envelope, ordered transformation describes change within it, threshold-dependent transition and self-sustaining dysregulation describe what occurs at its edge, and propagation extends the architecture across generations. It is the claim that one architecture meets them at both the organism and lineage scales that distinguishes the account.

Together they define the shape of an adequate account. Whether such an account can be developed is the question pursued in what follows: Section 5 examines the molecular evidence, Section 6 develops a systems model addressing these requirements, and Section 7 states the hypothesis that emerges from it.

## 5 The molecular architecture of living systems

Five bodies of molecular evidence indicate that architectures meeting these requirements are present in living systems, and that their disruption has recognisable consequences for evolutionary coherence at the lineage scale and for within-organism coherence, whose breakdown is observed in entrenched systemic disease.

The five architectures considered are representative of a broader class of organisational features rather than exhaustive. In each case the evidence is drawn from mainstream molecular and developmental biology; what is new is the lens through which it is considered. The order is significant: the first two sit in domains where higher-order organisation is already characterised under the systems-biology banner, while the remaining three (germline regulation, repetitive DNA control, centromere identity) sit in domains traditionally read through population-genetic, sequence-evolutionary, and molecular-genetic frameworks rather than as architectural organisation. Their inclusion is a substantive claim, not a stylistic choice: that the same architectural character operates not only where systems biology has characterised it, but at the molecular sites that condition lineage continuity.

### 5.1 The hierarchical organisation of developmental gene regulatory networks

Gene regulatory networks (GRNs) are not flat collections of interactions but hierarchically structured systems in which different components exhibit markedly different evolutionary behaviour. Davidson and Erwin (2006) identified a class of deeply embedded regulatory subcircuits, termed kernels: small numbers of transcription factors linked by recursive positive-feedback interactions, which stabilise core aspects of body-plan organisation across large phylogenetic distances. Their stability depends on all components being present together, an instance of what Wimsatt (1986) terms generative entrenchment: the more a system’s generated structure depends on a component, the more conserved and resistant to modification it becomes. Alterations to these kernels are almost invariably catastrophic, and they have remained essentially unchanged across hundreds of millions of years of evolution. The components above the kernel are more readily modified: intermediate sub-circuits are the locus of the regulatory changes associated with major body-plan reorganisation, while peripheral components account for finer-grained morphological variation between related lineages (Erwin and Davidson, 2009; Peter and Davidson, 2011).

The homeotic (Hox) gene clusters are the most fully characterised instance of this organisation. The Hox genes specify position along the anterior–posterior body axis and are conserved across the bilateria, exhibiting spatial collinearity — the order of the genes along the chromosome matching the order of the body regions they specify — with temporal collinearity in vertebrates additionally ordering activation in time (Kmita and Duboule, 2003). Their disruption produces homeotic transformation, the replacement of one body-region identity by another: a qualitative substitution of outcome, not a graded reduction. Collinearity is not universal, however: cluster organisation ranges from the tightly ordered vertebrate arrangement to disorganised, split, and even fully atomised clusters (Duboule, 2007), yet the anteroposterior order of expression can persist even where the physical cluster has disintegrated, as in the larvacean Oikopleura (Seo et al., 2004). What is conserved is thus the regulatory architecture that patterns the body axis, not the collinear arrangement in which it is most conspicuously realised: as with centromere identity, described below, the organisation is inherited while the substrate that realises it varies. The cluster itself arose by duplication and subfunctionalisation, its modifications tracking major transitions in body-plan organisation (Pearson, Lemons, and McGinnis, 2005); the architecture has a history, one instance of the emergence of components that Section 7 takes up as an open question.

This hierarchical organisation bears directly on the requirements set out in Section 4: it sustains long-term evolutionary coherence in body-plan organisation, because the kernels resist modification and their conservation is what allows the body plan to persist across deep time; it permits ordered transformation without collapse, because the levels above the kernel remain modifiable; and it constrains the forms of transformation, since the trajectories available to a lineage are shaped by the GRNs it already possesses. The stasis-and-transition pattern in the fossil record is consistent with this logic: with the kernels fixed throughout, stasis reflects conservation of the intermediate components and transition their modification.

Disruption of this hierarchical organisation has recognisable consequences in health-relevant contexts. The same regulatory architectures that stabilise body-plan development within a lineage are those whose disruption underlies several classes of developmental pathology, and whose aberrant reactivation is implicated in oncogenic transformation, where embryonic regulatory states are expressed outside their normal contexts (Kelleher et al., 2006; Aiello and Stanger, 2016). Such findings indicate that the integrity of hierarchical regulatory organisation is not only a feature of evolutionary stability but also a condition of organism-level coherence, and that its disruption is associated with characteristic forms of breakdown rather than undifferentiated dysfunction.

### 5.2 The bow-tie architecture of physiological regulation

A recurrent pattern across physiological networks is the bow-tie architecture (Csete and Doyle, 2002; 2004): a wide fan-in of diverse inputs, converging through a narrow conserved core of central processing, and a wide fan-out into diverse outputs. The narrow core is deeply conserved, intolerant of perturbation, and catastrophic when disrupted, while peripheral components are extensively modified across lineages and account for much of the variation observed between organisms.

The metabolic case is the most fully characterised. The conserved core (the reactions of glycolysis, the citric acid cycle, and the pentose phosphate pathway) is essentially invariant across the tree of life, traceable as a single inheritance to the last universal common ancestor (Weiss et al., 2016; Xavier et al., 2021), while the diverse anabolic and catabolic pathways that fan in and out of it are extensively modified across lineages (Csete and Doyle, 2004; Tanaka et al., 2005). The pattern is structurally analogous to the kernel-and-periphery organisation of developmental GRNs, realised at the scale of physiological rather than developmental regulation.

The same architecture has been characterised across functionally unrelated physiological domains: signal transduction (Friedlander et al., 2015), the innate and adaptive immune systems (Oda and Kitano, 2006; Kitano and Oda, 2006; Tieri et al., 2010), the integrated stress response (Pakos-Zebrucka et al., 2016; Costa-Mattioli and Walter, 2020), and circadian regulation (Takahashi, 2017; Patke et al., 2020). In each, the conserved core is where coherent function is sustained, and — like a GRN kernel — it operates as a coupled whole, so that its disruption produces loss of integrated function rather than graded decline.

When the bow-tie core is disrupted, characteristic pathologies follow. The metabolic dysregulation of cancer, the cumulative loss of metabolic flexibility in age-related decline, and the entrenched alternative regimes of chronic metabolic disease all involve disturbances at or near the bow-tie core of central metabolism (López-Otín et al., 2013; DeBerardinis and Chandel, 2016; Pavlova et al., 2022). Analogous consequences follow disruption of the conserved core in the other domains characterised above: autoimmune dysregulation of antigen presentation (Ishina et al., 2023), chronic inflammatory activation of NF-κB signalling (Mao et al., 2025), and dysregulation of the integrated stress response (Costa-Mattioli and Walter, 2020).

### 5.3 Regulatory systems operating in the germline

The germline hosts distinctive regulatory architectures. Unlike somatic processes, whose products are directly exposed to phenotypic selection, germline processes are partially insulated from such selection, yet they exhibit highly organised and conserved regulatory behaviour whose integrity is essential for the ordered transmission of biological organisation across generations.

Three features of germline regulation have been particularly well characterised. First, epigenetic states undergo systematic reprogramming in a coordinated cycle: parental epigenetic marks are largely erased, and new marks are established according to characteristic spatial and temporal patterns, ensuring that descendant cells inherit a regulatory configuration capable of supporting development rather than a residuum of the parental somatic state (Reik and Walter, 2001; Hackett and Surani, 2013). What is conserved across generations in this cycle is not the inventory of marks themselves but the architecture and machinery of reprogramming, through which the regulatory state appropriate to each generation is re-established. Second, transposable elements are regulated by a dedicated surveillance system centred on the piRNA pathway, which targets active elements with sequence specificity and maintains the integrity of the germline genome across generations (Aravin et al., 2007; Czech and Hannon, 2016). Third, the regulatory control of recombination, meiotic pairing, and chromosomal segregation ensures that the reorganisation of genetic material during meiosis occurs within structured constraints rather than as uncoordinated disturbance.

These systems bear on the requirements of Section 4: they maintain coherence across generations despite continual perturbation, permit ordered transformation within architectures that prevent collapse, and fail in threshold-dependent fashion: when the piRNA pathway is disrupted, transposable elements are derepressed and germline function fails in a cascading rather than gradual manner (Malone and Hannon, 2009). They are themselves transmitted: each must be present and operating in each generation for the next to be produced.

The piRNA pathway is commonly read as an evolved host defence against transposable-element parasitism, host genome and mobile elements engaged in a coevolutionary arms race: a reading that brings within the standard framework a finding it did not predict, that genomes tolerate large amounts of mobile-element activity without lineage collapse. The arms-race framing accounts for the silencing machinery but not for the finding that transposable elements are pervasively regulatory (Chuong et al., 2017). This the standard framework tried to re-accommodate by treating these elements as selfish sequences later co-opted for host functions. The architectural account reads the same phenomena differently: the pathway is part of the regulatory architecture through which the germline sustains the conditions of viable transmission. Its conservation, its threshold-dependent failure under partial compromise, and the structured way it channels transposable-element activity rather than merely suppressing it are intelligible as features of architectural organisation, not as products of a host–parasite arms race nor of the opportunistic recruitment of a parasite for regulatory use.

Germline architectures are the principal site at which the maintenance of regulatory organisation across generations is sustained, and they therefore provide the clearest case in which the integrity of evolutionary-coherence-sustaining architecture is visibly at stake. Architectures of a similar kind (epigenetic stabilisation of cell identity, surveillance of genomic integrity, and control of repetitive and mobile elements) also operate in somatic tissues, and their failure in those contexts produces loss of cellular identity, genomic instability, and the emergence of self-sustaining proliferative states characteristic of cancer (Zoghbi and Beaudet, 2016; Burns, 2017).

### 5.4 Genome dynamics and the regulation of repetitive DNA

The dynamics of repetitive DNA — transposable elements, satellite DNA, and tandem repeats — form a further body of evidence. Such sequences constitute a substantial fraction of most eukaryotic genomes, often a majority, and were so unexpected on the standard framework’s gradualist commitments that they were originally labelled junk DNA (Ohno, 1972), a designation that placed them outside the framework’s explanatory scope. The junk framing treated their persistence as material that selection had not eliminated rather than as evidence the framework needed to engage. The selfish-DNA account (Doolittle and Sapienza, 1980; Orgel and Crick, 1980) was a subsequent response that brought the sequences back within the standard framework by proposing selection at the level of the genetic element rather than the organism.

Subsequent research has revealed that much of this repetitive sequence has substantive regulatory function, undermining both the junk and the selfish-DNA framings in their original forms. Transposable elements undergo coordinated episodes of activity and suppression; their mobilisation is constrained by surveillance systems and by the chromatin contexts in which they are embedded; and their activity contributes to regulatory innovation, influencing chromatin organisation and the generation of new regulatory interactions (Chuong et al., 2017; Bourque et al., 2018). Satellite DNA exhibits its own patterns of expansion and contraction, its organisation implicated in heterochromatin structure and centromeric function. Across the genome, repetitive elements appear to be components of a dynamic regulatory system in which variability and control are closely coupled (Shapiro, 2011).

This coupling has direct relevance to the requirements of Section 4. It provides a substrate for ordered transformation: transposable element activity is a source of heritable change, but the variation it generates is constrained by surveillance systems that regulate it. It exhibits characteristic modes of breakdown: loss of control over transposable elements produces genomic instability, and chronic derepression is associated with a range of pathological states, including certain cancers, autoimmune conditions, and age-related decline (De Cecco et al., 2019; Gorbunova et al., 2021). It therefore illustrates how a single regulatory architecture can support both coherent function and, when disrupted, characteristic forms of dysregulation. On the present account these elements are among the established molecular processes through which the developmental programmes undergo ordered transformation; their now-recognised regulatory roles and their prevalence are thus one phenomenon rather than two to be reconciled.

The architectural account proposed here treats these phenomena differently from either the junk framing or the selfish-DNA account: the dynamics of repetitive elements are read as channelled through regulatory organisation whose conservation across lineages is what makes the persistence of the elements intelligible. That organisation is the conserved core of this architecture: the PIWI-protein surveillance machinery and its transposon-silencing function are deeply conserved across animals, even as the sequences they control, and the routes by which the guiding RNAs are produced, diverge widely (Ozata et al., 2019). This is conserved control over a variable substrate, the same pattern of conserved organisation and divergent realisation seen in the other architectures.

### 5.5 The maintenance of centromere identity

The centromere, the chromosomal region responsible for proper segregation during cell division, provides a further case. Centromeric DNA sequences are among the most rapidly evolving regions of the eukaryotic genome, diverging substantially even between closely related species, yet centromere function is highly conserved. This combination of rapid sequence divergence alongside conserved function has been termed the centromere paradox (Henikoff et al., 2001; Malik and Henikoff, 2009).

The centromere-drive account (Henikoff et al., 2001; Malik and Henikoff, 2009) explains the rapid sequence divergence as the outcome of conflict between centromeric DNA and meiotic machinery. Its scope is limited, however: rapid centromere evolution is documented in lineages that afford no opportunity for drive, including *Saccharomyces* yeasts, whose symmetrical meiosis excludes it, yet whose centromeres are among the fastest-evolving regions of the genome (Bensasson et al., 2008). Conserved function alongside rapid divergence thus persists where drive cannot operate.

The resolution of the paradox lies in centromere identity not being specified primarily by DNA sequence. It is maintained through epigenetic and structural mechanisms, including the incorporation of a specialised histone variant (CENP-A in most eukaryotes) and the assembly of a characteristic chromatin state that is propagated across cell divisions through self-templating feedback (McKinley and Cheeseman, 2016).

Centromere identity is therefore a particularly clear instance of architectural propagation: it is sustained not by conservation of the underlying sequence but by self-templating chromatin states transmitted across cell divisions, with the architecture partially decoupled from the sequence on which it is built. Function is conserved because the sequence is not what specifies it: the conserved-function-across-divergence pattern is a feature of architectural propagation, which the conflict account is not positioned to explain.

Breakdown of centromere identity has characteristic consequences: chromosome mis-segregation, aneuploidy, and genomic instability, all of which are associated with developmental pathology, infertility, and cancer (Barra and Fachinetti, 2018). Divergence of centromeric architecture between lineages also contributes to hybrid incompatibility: the same architecture whose within-organism breakdown produces the pathologies just described fails, at the lineage scale, to reconstitute coherently across a lineage boundary. This is the evolutionary-scale counterpart of within-organism dysregulation: dysregulation expressed not in the individual but in the failure of the architecture to reproduce itself coherently across generations.

### 5.6 What the evidence suggests

Across these five cases, diverse mechanisms instantiate a common organisation with the properties set out in Section 4 (Table 1). The germline is privileged as the conduit through which this organisation is propagated across generations and made available to development and soma each generation. That these systems are conserved and constrain the variation available to a lineage, and exhibit hierarchical, feedback-rich, and threshold-dependent organisation, is well supported above. That they are, further, expressions of a single common architecture rather than distinct systems sharing organisational features is the inferential step the argument proposes; the case for it rests on conservation by descent, developed in the Discussion, while the predictions of Part II test the architecture so inferred against gradient-selection and component-level alternatives.

**TABLE 1:** The five molecular architectures of Section 5 mapped against four of the requirements set out in Section 4: maintenance, ordered transformation, threshold-dependent transition, and propagation. The fifth requirement, self-sustaining dysregulation, is a systems-level property developed in the systems model (Section 6) and in Part II, rather than a per-architecture feature.

Molecular architecture	Maintains coherence by	Permits ordered transformation through	Characteristic failure mode	Role in propagation
Hierarchical GRN kernels	Conservation of deeply embedded regulatory subcircuits stabilising body-plan organisation across deep time	Modification of peripheral and intermediate components while the kernel is preserved	Catastrophic, not graded: developmental pathology; aberrant reactivation in oncogenesis	Re-deployment of conserved kernels in the development of each generation
Bow-tie physiological regulation	Conservation of a narrow core integrating diverse inputs and outputs (clearest in central metabolism)	Reconfiguration of peripheral pathways while the conserved core is preserved	Threshold-dependent transition to alternative regimes: metabolic and inflammatory dysregulation; loss of homeostatic capacity	Core architecture maintained across cell divisions and reconstituted each generation
Germline regulatory systems	Coordinated reprogramming, transposable-element surveillance, and meiotic regulation sustaining the conditions of viable transmission	Regulated reprogramming through which generation-appropriate regulatory states are re-established	Cascading germline failure on disruption of central components; genomic instability	Principal conduit for cross-generational propagation of the architecture
Repetitive-DNA regulation	Surveillance architectures channelling transposable-element activity rather than suppressing it outright	Constrained mobile-element activity as a source of regulatory innovation	Genomic instability; chronic derepression linked to cancer, autoimmunity, and ageing	Maintenance of regulated coupling across germline and somatic lineages
Centromere identity	Self-templating chromatin states propagated across cell divisions independently of DNA sequence	Tolerance of rapid sequence divergence without loss of function	Chromosome mis-segregation, aneuploidy, and genomic instability; contribution to hybrid incompatibility	Structural (non-sequence) propagation of chromosomal architecture across cell generations

It may be objected that systemic disease is a within-organism phenomenon and should be associated with the breakdown of within-organism regulation, not with evolutionary coherence. But the two are not separate. The architectures whose disruption is observed in disease are the same architectures whose conservation sustains biological coherence: a GRN kernel conserved across hundreds of millions of years has a lineage-level and a within-organism aspect, not two separate existences. A within-organism account correctly identifies what fails. What it cannot supply on its own is why failure converges across conditions of unrelated proximate cause: a convergence intelligible only through the evolutionary depth of the architecture that has failed. The breakdown observed in disease is in every case this within-organism aspect; at the lineage scale the disruption of so deeply conserved a core is not observed, because a lineage that lost it would not persist: the ordinary survivorship condition of evolutionary evidence, noted above for transformation and applying equally to the conserved cores here.

The maintenance of this organisation is itself the condition of viable transmission across deep time. The account is not a redescription immune to evidence: as set out above, it commits to discriminating predictions, developed fully in Part II, that could disconfirm it. Table 1 maps these architectures against the requirements of Section 4.

## 6 A systems model of the coherence-sustaining architecture

Drawing on Section 5, the model treats the five architectures, there considered separately, as expressions of a single higher‐order regulatory architecture realised through different mechanisms at different sites: the architecture sustaining biological coherence.

Three features define the model: an operating envelope, a bounded region of system states within which coherence is sustained and ordered transformation accommodated at both the lineage and organism scales; a set of dynamics at the boundary of that envelope, by which the architecture responds to perturbations approaching or exceeding its limits; and propagation across generations, principally through the germline. Each is developed below.

### 6.1 The operating envelope

Within its operating envelope, the architecture sustains biological coherence at both scales through three closely connected properties. It maintains the conditions of coherent organisation despite continual perturbation from internal noise, component-level variation, and the variation of environments across generations. It is robust: departures from characteristic states are actively countered, and the architecture returns toward conditions in which coherent function is sustained. And it accommodates ordered transformation: changes in component activity, in the abundance or identity of component populations, and in the configuration of regulatory subsystems can occur without threatening the coherence of the system as a whole, whether lineage or organism.

What makes these properties possible, on this account, is not the conservation of particular components but the organisation of the architecture within which those components operate. Each of the architectures examined in Section 5 illustrates the principle: in each case, the architecture actively sustains a space of permitted states (the operating envelope) within which ordered transformation occurs and beyond which coherence is no longer sustained.

The envelope is not static. Its dimensions are shaped by the state of the architecture itself, which may be altered by developmental stage, physiological context, age, or accumulated perturbation. An architecture whose regulatory organisation is compromised, whether through mutational degradation, chronic stress, or loss of surveillance, may operate within a narrower envelope than an intact one and may therefore be more susceptible to transitions out of coherent function.

### 6.2 Dynamics at the boundary

The dynamics of the architecture at the boundary of its operating envelope differ qualitatively from its dynamics within the envelope. Within the envelope, perturbation is absorbed and coherence is preserved. At and beyond the boundary, behaviour becomes threshold-dependent: small additional perturbations can produce qualitatively different outcomes, and the architecture may undergo changes of regime rather than quantitative adjustment within the existing one. Figure 1A illustrates these characteristic dynamics schematically.

**FIGURE 1:**
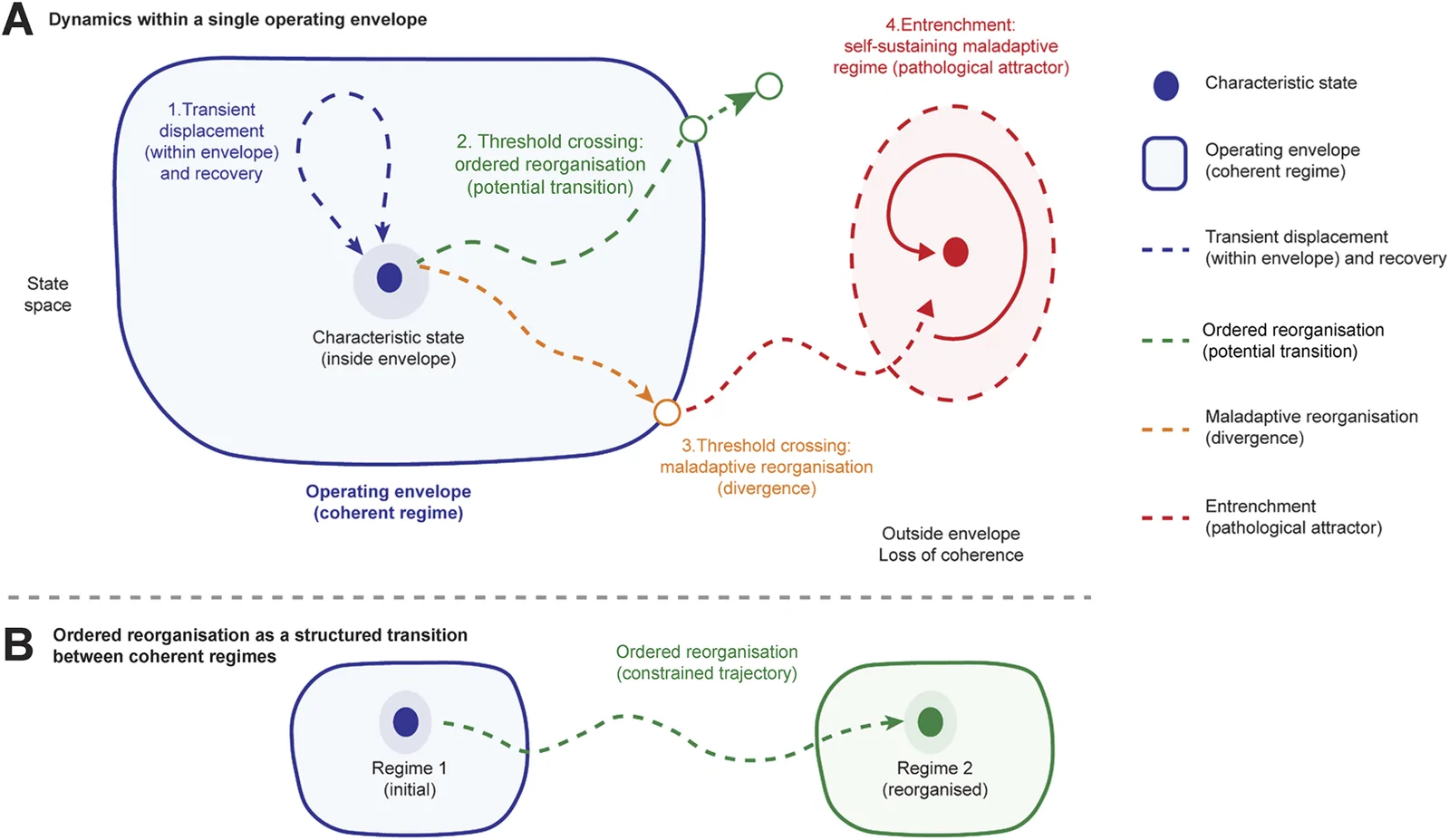
The operating envelope and the characteristic dynamics of a regulatory architecture. The operating envelope is the region of state space within which the architecture maintains coherent organisation; crossing its boundary is a qualitative transition rather than a graded perturbation. **(A)** Within a single operating envelope, a perturbation from the characteristic state can produce transient displacement followed by recovery, in which the architecture remains within the envelope; or it can cross the envelope boundary into one of three transitions: ordered reorganisation, maladaptive reorganisation, or entrenchment in a self-sustaining maladaptive regime. **(B)** Ordered reorganisation shown as a structured transition between coherent regimes: the architecture leaves one envelope and settles into another along a constrained trajectory, preserving coherence at a higher level. The state space is conceptual and the figure is a schematic rather than a phase portrait of a particular system. The dynamics shown are those of the within-organism scale, over the lifespan; the same architecture, conserved across deep time, is what sustains evolutionary coherence at the scale of the lineage.

Three kinds of transition are especially important. The first is transient displacement. Here the architecture crosses into a region of reduced coherence, but its underlying integrity remains intact, and it returns to its operating envelope once the perturbation passes. This is the familiar pattern of physiological recovery from stress or developmental correction following early disturbance.

The second is ordered reorganisation. Here the architecture undergoes a structural transition in which the organisation itself is modified, but in a way that preserves, at a higher level, the coherence of the system undergoing it (Figure 1B). Developmental stage transitions, immune maturation, and the more extensive reorganisations associated with evolutionary transitions across lineages are instances of this kind. The architecture is not merely restored; it is transformed in a way that preserves continuity through the transformation. This is also how new architecture arises: not imported from outside the system, but produced through the reorganisation of existing developmental and physiological architecture into new coherent regimes. The reorganisation is realised through a wide set of established molecular processes, of which transposable-element activity is the most prominent, treated in detail in the companion paper on speciation. Generation is in this sense internal to the lineage rather than imported from outside it: new architecture is a transformation of existing architecture, effected through these processes, not the product of constraint-breaking chaos.

The third is maladaptive reorganisation. Here the architecture undergoes a transition into a regime in which the coherence of the prior state is lost, and the resulting state is either unstable or stable in a pathological rather than functional sense. The distinction between ordered and maladaptive reorganisation is central: both involve changes in organisation rather than perturbations within an existing regime, but they differ in whether the transition preserves coherence at a higher level or produces a state that is dysfunctional, unstable, or pathological. What determines which outcome obtains appears to depend on the integrity of the regulatory systems through which change proceeds, and where those systems are compromised, the same kind of perturbation that might otherwise yield controlled reorganisation may instead produce dysregulation. This is where the model becomes directly relevant to systemic health.

### 6.3 Entrenchment

A particularly important feature of the model concerns what happens after a maladaptive transition has occurred. Not all such transitions are self-limiting. In some cases, the regulatory feedbacks that normally stabilise coherent function come instead to stabilise the dysregulated state: the same processes that would, under normal conditions, return the architecture to its operating envelope now hold it in a pathological regime. Return to prior function becomes difficult or effectively impossible even when the initiating perturbation has passed.

This is one of the model’s central explanatory commitments: the same integrative and stabilising properties that make the architecture robust under normal conditions (feedback, redundancy, coordinated regulation) are what allow dysregulated states, once entered, to become self-sustaining.

Oncogenic transformation, in which regulatory feedbacks that normally sustain differentiated identity come to sustain a self-reinforcing proliferative state, is one instance. Certain autoimmune conditions, in which the processes that normally maintain tolerance come instead to maintain chronic inflammatory activation, provide another. Entrenched metabolic states characteristic of several chronic conditions, and some forms of cumulative developmental or physiological dysregulation, exhibit the same general pattern.

What the model offers here is not a mechanism for any particular pathology but a characterisation of why such entrenchment is possible at all: because the same feedback structures that stabilise coherent function can, when the architecture is displaced sufficiently far, stabilise an alternative regime. Stability and pathology are therefore not opposites in organisational terms but alternative expressions of the same regulatory capacities under different conditions. Part II develops this account of entrenchment into a discriminating prediction and draws out its implications for systemic health, including the conditions under which entrenched pathological regimes can be reversed. Figure 1A depicts the entrenched regime as a self-sustaining attractor in state space, displaced from the operating envelope and stabilised through the same feedbacks that otherwise sustain coherent function.

### 6.4 Propagation

The final feature of the model is propagation across generations. The higher-order regulatory architecture is not merely present in each instantiation of the lineage; it is transmitted such that descendant systems inherit the conditions under which evolutionary coherence, ordered transformation, and resistance to dysregulated entrenchment can be re-established in each new generation. What is propagated is the architecture and the conditions of its execution, not a fixed inventory of regulatory states.

This is where the germline enters the model most directly. Germline regulatory systems (epigenetic reprogramming machinery, piRNA-mediated transposable element surveillance, the regulatory organisation of meiosis, and the maintenance of centromere identity and chromosomal structure) are the principal conduit through which the architecture is propagated. Their integrity is therefore a condition of the continued expression of the model’s properties in descendant systems.

Propagation is not a separate principle added from outside; it is the across-generation expression of the same architectural logic that operates within organisms. The architecture that sustains evolutionary coherence within a generation is, in the germline context, what sustains the transmission of the conditions for coherence across generations. Disruption of germline organisation therefore has consequences that mirror, at the cross-generational scale, those of disrupting regulatory organisation within an organism, propagating architectural compromise to descendant systems. What is at stake is the integrity of the architecture itself across generations, rather than the transmission of specific regulatory states intact. This cross-generational failure is the evolutionary-scale counterpart of within-organism dysregulation, and its clearest concrete instances — the Mortal Germline phenotype and structured transgenerational perturbation — are developed in Part II.

## 7 The CHORA hypothesis

The hypothesis advanced here is that living systems possess, as a feature of their organisation, a higher-order regulatory architecture whose integrity sustains biological coherence, whose operation accommodates ordered transformation, and whose disruption underlies characteristic modes of breakdown. We refer to this architecture as a Common Higher-Order Regulatory Architecture, or CHORA (pronounced KOH-rah), and to the hypothesis that it operates as described as the CHORA Hypothesis.

It is worth being explicit about what kind of thing this architecture is. The architecture is not a single structure occupying a fixed location in the cell or genome. It is a common organisational character, largely conserved, realised through different mechanisms at different sites: the systems examined in Section 5 — hierarchical gene regulatory networks, bow-tie physiological regulation, germline regulatory systems, the regulation of repetitive DNA, and centromere identity — are its expressions, not independent phenomena that happen to share organisational features. In each, a conserved core carries this character, and is propagated across generations, principally through the germline. It is one architecture for two reasons. One organising character is realised across their physically diverse cores. And in each, that character is a single existence across two scales, not two separate ones: the conservation that produces coherent organisms in real time is the same conservation that, inherited across the lineage, sustains it over deep time. And in each, the transformation of the other levels is not merely constrained, as developmental constraint describes, but regulated: by the molecular processes now known to regulate gene expression, within the organism and across evolutionary divergence alike (ENCODE Project Consortium, 2012; Chuong et al., 2017). The architecture is the conserved higher-order organisation these regulatory processes realise and through which they sustain coherence, active as an organised whole, not a further agency that directs them. These processes, transposable-element activity foremost among them, are its molecular signatures, the sites at which it can be observed at work.

A definition of this kind can seem unsatisfactory: it does not say, concretely, what the architecture is. That dissatisfaction is the appropriate response to an entity inferred from a convergent explanatory residual rather than characterised directly, and it is not peculiar to this framework. The repetitive sequence in which much of the architecture is realised has itself been called the ‘dark matter of the genome’, though first for its inaccessibility to sequencing rather than any explanatory role (Sedlazeck et al., 2018); the analogy drawn here is of a different and more exact kind. Dark matter, in physics, is defined by the motions it must account for, not by any direct characterisation of its nature, and it remains in just this sense unsatisfactory: no one can yet say what it is. The parallel is to this epistemic status. What keeps such a posit within science, and distinguishes it from the vital principles of an earlier biology, is twofold. The first is the explanatory power set out above: a unifying and parsimonious account of phenomena the standard view addresses only piecemeal. The second is discipline by evidence. Unlike principles that explained everything and predicted nothing, the architecture commits to discriminating predictions by which it can be tested, and fail. Its intrinsic nature, what kind of thing has this causal standing, is left open here, and is the subject of a companion paper on the framework’s foundations.

### 7.1 What the hypothesis commits to

Four claims define the hypothesis.

The first is that the higher-order regulatory architecture operates as an integrated level of organisation across cells, tissues, organisms, and generations. This integration is itself hierarchically structured: within each architecture, some components occupy an architecturally central position, whose disruption threatens the coherence of the system as a whole, while others occupy more peripheral positions, whose modification is comparatively well tolerated. The architectures examined in Section 5 are treated as expressions of a common organisational character realised through different mechanisms at different sites, rather than as independent phenomena that happen to share organisational features.

The second claim is that the germline occupies a specifically privileged position in the propagation of the architecture. The germline regulatory systems examined in Section 5 are the principal conduit through which this architecture persists across generations, with the regulatory configuration appropriate to each generation re-established through the architecture transmitted from the previous one rather than through the inheritance of fixed regulatory states. The architecture is also expressed and sustained in somatic tissues, and its disruption in those tissues underlies much of what the hypothesis identifies as systemic breakdown; but the expression of the architecture in somatic contexts depends on its prior transmission through the germline.

The third claim is that the same architectural features that sustain biological coherence also underlie the characteristic modes of its failure. Robustness, ordered transformation, and the conditions of breakdown are consequences of the same architectural logic operating under different conditions, rather than independent phenomena to be explained separately. The hypothesis therefore offers a unified framework for the phenomena treated within separate disciplinary domains in the introduction and Section 2.

The fourth claim is that disruption of the architecture at the organism level underlies the convergent signatures of systemic disease. The same architectures that sustain evolutionary coherence over the lineage produce, when disrupted at the organism level, the characteristic forms of pathological breakdown: loss of hierarchical regulatory constraint, inappropriate activation of context-restricted regulatory states, and the entrenchment of dysregulated regimes through repurposed feedback.

### 7.2 What the hypothesis does not claim

Clarity about what the hypothesis does not claim is equally important.

It does not posit a new force or substance. To characterise the architecture by its explanatory role is not to add a vital principle animating living matter.

It does not claim that selection plays no role in biological change. Selection acts on the variation the architecture makes available, on the phenotypic outputs it produces, and on the relative success of lineages exhibiting different expressions of its operation. The framework treats selection and architecture as distinct concerns at different levels: selection bears on the dynamics of variation and adaptation across generations; the architecture bears on the organisation that sustains evolutionary coherence over deep time and within which selection operates. The two are complementary rather than competing: the framework articulates the architectural level rather than displacing the selectionist account of variation and adaptation.

It does not take a position on the levels-of-selection question. In referring to the relative success of lineages, the framework does not commit to selection acting at the level of the lineage, the species, or the clade, nor does it adjudicate between single-level and multilevel accounts of how selection operates. The architecture it characterises is the organisation that any selective process presupposes, the conditions under which heritable, persistent, selectable units exist at all, and is compatible with monist and multilevel accounts alike.

It does not claim to provide mechanisms for particular diseases or particular evolutionary transitions. The hypothesis is a framework within which such mechanisms might be situated, not a substitute for the specific mechanistic accounts that particular phenomena require. The discriminating predictions developed in Part II indicate the observations that bear on the framework’s adequacy; engagement with specific pathologies and evolutionary transitions remains the work of the relevant research literature.

Its focus is the eukaryotic and especially metazoan biology from which the evidence is drawn, for the reasons of scope set out in the Introduction; this focus is not a claim that architectures of CHORA-like character are confined to that domain. Its components are in fact of differing antiquity: the conserved metabolic core traces to the last universal common ancestor and is shared across all cellular life (Section 5), while the elaborated developmental and germline architectures on which the disease link depends are eukaryotic and metazoan. The framework may therefore extend beyond the domain treated here, and the emergence of its different components at different depths of descent is a question its architectural framing opens rather than closes.

## 8 Discussion

The case for the architecture is abductive, in the manner set out in the Introduction: beginning from recurrent patterns and asking what form of organisation would render them intelligible. The preceding sections assemble independent bodies of evidence — developmental, physiological, germline, chromatin, and disease — not obviously connected, yet pointing together to the same conclusion. Their best explanation is not a series of separate conservation mechanisms but a single inherited higher-order architecture, of which the five patterns are expressions. The inference draws its force from two features of that explanation: the consilience of these independent lines of evidence (Whewell, 1840), and its parsimony: one posit in place of the several independent auxiliary hypotheses the standard view requires, each accommodating one pattern after the fact.

This architecture is not a separate causal force acting alongside the molecular processes, nor an inert pattern they leave behind, but the higher-order organisation in and through which they sustain coherence, active as an organised whole, though not as an agency directing its parts. These established molecular processes are its molecular signatures: the observable form in which the architecture works. A precedent: Dover (1982) proposed molecular drive, the propagation of repetitive sequence through a lineage by genomic turnover, as a directional process distinct from selection and drift, capable of establishing novelty independently of selection on phenotypic outputs. Dover’s account concerned homogenisation, the concerted evolution by which the members of a repeated-sequence family are kept uniform across a lineage; the regulatory roles of these elements, and their contributions to chromatin and centromere function, were characterised largely after his work (ENCODE Project Consortium, 2012; Chuong et al., 2017). The processes that transform the developmental programmes are of this same non-selective, mechanistic kind, operating not on isolated sequences but on the organisation those sequences realise. That transformation falls on the modifiable levels of the developmental gene regulatory networks Peter and Davidson (2011) characterised, while the conserved kernels are preserved, so that the higher-order architecture is maintained across generations even as its lower levels change.

Transposable-element activity is touched upon here and further elaborated in the companion paper on speciation; the molecular drive underlying concerted evolution and chromosomal rearrangement are developed there. That such processes should yield coordinated, architecture-preserving outcomes rather than dissipative change was not explained when molecular drive was first proposed, and is not taken up here or in the companion paper on speciation, which describes the processes that conserve and transform the architecture. Why the outcome is ordered rather than dissipative is the deep question reserved for the companion paper on the framework’s foundations.

### 8.1 A single architecture, or convergent solutions?

A reasonable objection is that the five architectures share organisational features — hierarchy, a conserved core, threshold dynamics, feedback — without this establishing that they are expressions of one architecture rather than independently evolved solutions to similar problems. The hypothesis is committed to more than shared organisational form.

What separates the two readings is conservation by descent. Were the recurrence convergent, the organisational similarity would be accompanied by independent molecular origins, as with circuit-level network motifs, which recur across networks without being inherited as units (Milo et al., 2002). The architecture described here is different, and the evidence of descent runs across the architectures of Section 5 in two forms. Where the units themselves are transmitted, descent is most direct: the gene regulatory network kernels are not merely similar across lineages but the same kernels, conserved across hundreds of millions of years and recalcitrant to modification (Section 5; Davidson and Erwin, 2006). Centromere identity makes the point more sharply, because here conservation cannot be attributed to sequence: its function is conserved across lineages while the underlying DNA sequence diverges rapidly, propagated by self-templating chromatin states rather than by the sequence on which it sits (Section 5; Henikoff et al., 2001; McKinley and Cheeseman, 2016). Where instead the regulatory organisation is conserved while the components it acts on turn over, what is inherited is the architecture rather than its parts: the germline and repetitive-DNA surveillance systems are transmitted whatever the turnover of the elements they regulate (Section 5; Aravin et al., 2007; Malone and Hannon, 2009), and the conserved core of the physiological bow-tie, central metabolism, is invariant across the tree of life and traceable to the last universal common ancestor (Section 5; Weiss et al., 2016; Xavier et al., 2021). A function conserved on a divergent substrate, sustained by transmission rather than sequence, is inherited, not reinvented, a signature convergence is not well placed to reproduce. The descent at issue runs within each architecture rather than between them: the five do not share a common genealogical origin, and the claim is not that they descend from one ancestral structure but that each independently exhibits, and transmits, the same organisational character. What is inferred is a shared organisation, not a shared lineage.

This yields a discriminating expectation with both postdictive and prospective force. The postdictive support is already in hand: the centromere paradox, deep homology across the bilaterian body plan, and the conservation of the same kernels across lineages with differing ecologies (Section 5) are established observations that the inheritance reading organises and the convergence reading does not readily accommodate. The prospective test is systematic: on the convergence reading, conservation should track selective pressures on outputs and be decoupled from descent; on the architectural reading, it should track descent and architectural centrality across lineages whose selective regimes differ. What distinguishes the hypothesis from the convergence alternative is therefore not that the features are shared but that they are inherited. This is a falsifiable condition: were the organisational core shown to be independently derived across lineages rather than transmitted through them, the hypothesis would fail. The condition follows directly from two of its four commitments, that the architecture operates as an integrated level across generations and that the germline is the principal conduit of its propagation, which jointly entail conservation by descent. Convergence is thus the alternative Part I addresses; the six predictions developed in Part II presuppose this inherited architecture and distinguish it further from a different set of alternatives: gradient-selection, genetic-inheritance, and component-level accounts. The six are summarised in Table 2.

**TABLE 2:** The six discriminating predictions of the series, grouped by the commitment from which each follows. The predictions are summarised here and developed and tested in Part II.

Commitment the prediction follows from	Discriminating prediction (developed and tested in part II)
Integrated operation of the architecture across cells, tissues, organisms, and generations	Catastrophic rather than graded failure at architecturally central components Concentration of lineage transitions at mid-hierarchical levels Bidirectional intergenerational consequences at germline–soma coupled components
Germline priority in propagation	Intergenerational erosion under partial germline compromise, with characteristic somatic analogues
The coherence–breakdown relationship	Entrenchment of pathological regimes through feedback architectures that also sustain coherent function
Disruption at the organism level	Convergence of distinct systemic pathologies on shared architectural signatures

### 8.2 Relation to genome architecture theory

An independently developed, system-level account that shares substantial ground with the present hypothesis is Genome Architecture Theory (GAT; Heng and Heng, 2022; Heng and Heng, 2023), which likewise locates the primary organisation of living systems above the gene. The two accounts converge on much: on system-level inheritance not reducible to gene sequence, on punctuated transformation, on disease as a system-level event, and on framing the system level as one of information (Table 3). This shared framing connects to an established information-theoretic treatment of regulatory systems, in which developmental gene regulatory networks encode and read out positional information (Peter and Davidson, 2017) and inheritance systems transmit information across generations (Jablonka, 2002); on the present account the conserved architecture is the active organisation of which that information is the heritable record, carried over deep time. GAT’s karyotype coding can be read as one candidate realisation of the system-level inheritance the present account requires, compatible with it without being identical.

**TABLE 3:** Genome Architecture Theory and the CHORA Hypothesis compared. Both frameworks locate the primary organisation of living systems above the gene; they differ in what they take the system level to be, how they understand its transformation, and their primary evidence base.

Point of comparison	Genome Architecture Theory (GAT)	The CHORA hypothesis
Locus of the system level	The karyotype: chromosomal order and number, proposed as the carrier of system-level inheritance (“karyotype coding”)	Not a physical locus but a higher-order organisation, realised across gene regulatory networks, germline regulatory systems, chromatin, and centromere identity yet identical to none of them; characterised by what it does rather than by a carrier
Unit of inheritance	The whole genome/karyotype, rather than the gene, as the primary heritable unit	The regulatory architecture, propagated primarily through the germline, rather than any fixed inventory of states
Mechanism of transformation	Two phases distinguished by genomic level: macroevolution by karyotype change (chromosomal reorganisation), microevolution by gene mutation. Stress-induced genome chaos breaks existing constraints to generate new genome systems	The molecular processes of reorganisation (transposable-element activity, the molecular drive underlying concerted evolution, chromosomal rearrangement) operate within the regulatory architecture. Punctuation follows from the threshold dynamics of these systems: repetitive-sequence homogenisation holds a lineage’s form at a bounded equilibrium (stasis), and because such a system admits more than one stable configuration, perturbation past a threshold shifts it to an alternative state rather than moving it gradually, with transposable-element restructuring supplying organised novelty at the transition. The developmental hierarchy governs where body-plan modification is tolerated (kernels conserved, periphery modifiable), not why change is episodic. The full account is developed in a companion paper on speciation. Constraint-breaking is the breakdown regime, not the generative one
Generation of new architecture	Generated by stress-induced genome chaos; the reorganised karyotype is itself the new system	New architecture arises through ordered transformation of existing architecture (the row above), realised through established molecular processes rather than through constraint-breaking chaos. Generation is thus internal to the lineage: new architecture is a transformation of the old, effected by these processes
Primary evidence base	Somatic cell genetics, principally cancer cytogenetics (karyotype change across tumour phase transitions)	Developmental gene regulatory networks, germline regulation, physiological (bow-tie) organisation, and repetitive-DNA and centromere systems
Treatment of disease	Disease, paradigmatically cancer, as a genome-level macroevolutionary event: the creation of a new system	Entrenched disease as the breakdown regime of a conserved architecture: a maladaptive attractor reached when the operating envelope is exceeded
Organising principle	Information management: the self-creation, preservation, modification, and use of genomic information, with the creation of new system information through genome reorganisation as the driver of macroevolution	Ordered management of biological information: what the conserved architecture preserves, permits to vary, and transmits across generations. Emphasis falls on conservation and ordered transmission rather than creation, in the sense of established information-theoretic treatments of regulatory networks and inheritance (Peter and Davidson, 2017; Jablonka, 2002); selection retains its role at the level of the variation the architecture makes available
Relation to the Modern Synthesis	Explicitly rejects “macroevolution = microevolution + time”; the system level is primary over the gene	Does not reject selection: selection operates on the variation the architecture makes available, at a different level from the architecture itself. The two are treated as complementary rather than competing

They differ, however, at the most fundamental level, on the nature of transformation itself. GAT locates the system level in the karyotype and takes its mechanism of transformation to be stress-induced genome chaos, characterised chiefly in cancer; the present account locates it in regulatory architecture and treats large-scale reorganisation as ordered transformation channelled into organised novelty. This is not to leave the present account without a mechanism: the transformation it proposes is realised through established molecular processes, transposable-element activity paradigmatic among a wider set (Table 3), whose detailed treatment is developed in the companion paper on speciation. The two accounts thus differ not in whether transformation has a molecular basis but in what that basis is: where GAT emphasises the creation of new system information through chaotic reorganisation, the present account emphasises its ordered conservation and transmission. The opposition is fundamental: where GAT depends on chaos for its generative principle, the present account depends on ordered transformation; and what GAT documents as the chaotic source of new organisation is, on the present account, the regime of breakdown. The same contrast appears in how each account reads genome reorganisation: where GAT recruits mobile-element and repetitive-DNA activity as part of the constraint-breaking chaos from which novelty arises, the present account reads the same processes as ordered molecular drive, the means by which inherited architecture is transformed. What GAT treats as disorder, the present account treats as a mechanism of ordered transformation.

### 8.3 Testability, measurement, and falsification

Two further points concern what can and cannot presently be measured. The notions of biological coherence and the operating envelope are, at this stage, more readily characterised qualitatively than quantitatively: architectural centrality, coupling, and entrenchment do not yet have agreed operational measures with the precision available to, for instance, sequence-based measures of conservation. This is a genuine limitation, and the predictions above are framed to depend on qualitative contrasts (catastrophic versus graded failure, presence versus absence of a coupling mechanism, architectural versus component-specific convergence) that can be assessed without such measures, pending their development. The privileged role the hypothesis assigns to the germline should likewise be understood in architectural terms: it is privileged not as an additional inheritance channel alongside genetic and epigenetic mechanisms, but as the site at which the regulatory architecture is sustained across generations.

Finally, the claims linking architectural disruption to disease are deliberately scoped. The category is one of entrenched pathological regimes: self-sustaining dysregulated states of a largely intact architecture outside its envelope (Figure 1). Though not usually grouped together, these include entrenched chronic disease and most cancers, as outlined in Part II, and certain persistent infections developed in a companion paper on entrenchment. Not all disease is likely to be of this kind, and the framework claims only this category, not a general account of pathology. The claim is bounded by the evidence of Section 5 and the predictions of Part II, and offered as a candidate account, not an established mechanism.

### 8.4 Concluding remarks

This paper has advanced the CHORA Hypothesis as a candidate account of how biological coherence is sustained, transformed, and lost in eukaryotic and especially metazoan biology. By abductive inference from recurrent phylogenetic, developmental, and physiological patterns, it identifies a shared explanatory gap in current evolutionary and biomedical frameworks, specifies the requirements an adequate account must meet, and argues that the relevant molecular architectures constitute a single higher-order regulatory architecture.

The framework treats evolutionary coherence and systemic health as expressions of one architecture operating under different conditions. Health and entrenched systemic disease, on this account, are not opposing biological states defined by component function but alternative regimes of the same architecture. This is the framework’s central contribution to the Research Topic’s systemic health framing: that how living systems sustain coherent function over deep time and how they break down within a lifespan are expressions of one organisational phenomenon.

Part II develops the implications for the conceptualisation of disease, ageing, intergenerational health, and therapeutic logic.

The hypothesis has consequences beyond the domains addressed here and in Part II: for the relation between selection and the architectural conditions of its operation, for the kind of explanation appropriate to the persistence of organised biological function across deep time, and for the genealogy of organisational thinking within which the framework sits.

The question of how the architecture itself came to be is not separable from the origin of life, where systems-science approaches locate the threshold of living organisation in the emergence of self-maintaining boundaries (Laouris, 2026), and it touches ancient questions about the relation of organised form to material substrate: Plato’s Timaeus (48e–52 d; Plato, 1997) through the chora, the formless receptacle in which sensible form arises and which the framework’s acronym fortuitously echoes, and Aristotle’s Generation of Animals (Aristotle, 1942) through form as the principle of living generation. These conceptual foundations are developed in the companion paper on the framework’s philosophical basis. Whether the framework proves adequate is an empirical question, to be assessed against the predictions the hypothesis generates and the phenomena it seeks to integrate.

## Data Availability

The original contributions presented in the study are included in the article/supplementary material, further inquiries can be directed to the corresponding author.

## References

[B1] Aiello N. M. Stanger B. Z. (2016). Echoes of the embryo: using the developmental biology toolkit to study cancer. Dis. Models and Mech. 9 (2), 105–114. 10.1242/dmm.023184 26839398 PMC4770149

[B2] Alberts B. Heald R. Johnson A. Morgan D. Raff M. Roberts K. (2022). Molecular Biology of the Cell. 7th edn. New York: W. W. Norton and Company. ch. 20 (“Cancer”).

[B3] Aravin A. A. Hannon G. J. Brennecke J. (2007). The Piwi-piRNA pathway provides an adaptive defense in the transposon arms race. Science 318 (5851), 761–764. 10.1126/science.1146484 17975059

[B4] Aristotle (1942). Generation of Animals (c. 350 BCE). Editor A. L. Peck. Cambridge, MA: Harvard University Press (Loeb Classical Library).

[B5] Barabási A.-L. Gulbahce N. Loscalzo J. (2011). Network medicine: a network-based approach to human disease. Nat. Rev. Genet. 12 (1), 56–68. 10.1038/nrg2918 21164525 PMC3140052

[B6] Barra V. Fachinetti D. (2018). The dark side of centromeres: types, causes and consequences of structural abnormalities implicating centromeric DNA. Nat. Commun. 9, 4340. 10.1038/s41467-018-06545-y 30337534 PMC6194107

[B7] Barton R. A. Aggleton J. P. Grenyer R. (2003). Evolutionary coherence of the Mammalian amygdala. Proc. R. Soc. B Biol. Sci. 270 (1514), 539–543. 10.1098/rspb.2002.2276 12641910 PMC1691272

[B8] Bensasson D. Zarowiecki M. Burt A. Koufopanou V. (2008). Rapid evolution of yeast centromeres in the absence of drive. Genetics 178 (4), 2161–2167. 10.1534/genetics.107.083980 18430941 PMC2323805

[B9] Bourque G. Burns K. H. Gehring M. Gorbunova V. Seluanov A. Hammell M. (2018). Ten things you should know about transposable elements. Genome Biol. 19, 199. 10.1186/s13059-018-1577-z 30454069 PMC6240941

[B10] Burns K. H. (2017). Transposable elements in cancer. Nat. Rev. Cancer 17 (7), 415–424. 10.1038/nrc.2017.35 28642606

[B11] Chuong E. B. Elde N. C. Feschotte C. (2017). Regulatory activities of transposable elements: from conflicts to benefits. Nat. Rev. Genet. 18 (2), 71–86. 10.1038/nrg.2016.139 27867194 PMC5498291

[B12] Costa-Mattioli M. Walter P. (2020). The integrated stress response: from mechanism to disease. Science 368 (6489), eaat5314. 10.1126/science.aat5314 32327570 PMC8997189

[B13] Csete M. E. Doyle J. C. (2002). Reverse engineering of biological complexity. Science 295 (5560), 1664–1669. 10.1126/science.1069981 11872830

[B14] Csete M. E. Doyle J. C. (2004). Bow ties, metabolism and disease. Trends Biotechnol. 22 (9), 446–450. 10.1016/j.tibtech.2004.07.007 15331224

[B15] Czech B. Hannon G. J. (2016). One loop to rule them all: the ping-pong cycle and piRNA-guided silencing. Trends Biochem. Sci. 41 (4), 324–337. 10.1016/j.tibs.2015.12.008 26810602 PMC4819955

[B17] Davidson E. H. Erwin D. H. (2006). Gene regulatory networks and the evolution of animal body plans. Science 311 (5762), 796–800. 10.1126/science.1113832 16469913

[B18] De Cecco M. Ito T. Petrashen A. P. Elias A. E. Skvir N. J. Criscione S. W. (2019). L1 drives IFN in senescent cells and promotes age-associated inflammation. Nature 566 (7742), 73–78. 10.1038/s41586-018-0784-9 30728521 PMC6519963

[B19] DeBerardinis R. J. Chandel N. S. (2016). Fundamentals of cancer metabolism. Sci. Adv. 2 (5), e1600200. 10.1126/sciadv.1600200 27386546 PMC4928883

[B20] Doolittle W. F. Sapienza C. (1980). Selfish genes, the phenotype paradigm and genome evolution. Nature 284, 601–603. 10.1038/284601a0 6245369

[B21] Dover G. (1982). Molecular drive: a cohesive mode of species evolution. Nature 299 (5879), 111–117. 10.1038/299111a0 7110332

[B22] Duboule D. (2007). The rise and fall of hox gene clusters. Development 134 (14), 2549–2560. 10.1242/dev.001065 17553908

[B23] Eldredge N. Gould S. J. (1972). Punctuated equilibria: an alternative to phyletic gradualism. In: Schopf T. J. M. (ed.) Models in Paleobiology. San Francisco: Freeman. 82–115.

[B24] ENCODE Project Consortium (2012). An integrated encyclopedia of DNA elements in the human genome. Nature 489 (7414), 57–74. 10.1038/nature11247 22955616 PMC3439153

[B25] Erwin D. H. (2007). Disparity: morphological pattern and developmental context. Palaeontology 50, 57–73. 10.1111/j.1475-4983.2006.00614.x

[B26] Erwin D. H. Davidson E. H. (2009). The evolution of hierarchical gene regulatory networks. Nat. Rev. Genet. 10 (2), 141–148. 10.1038/nrg2499 19139764

[B27] Friedlander T. Mayo A. E. Tlusty T. Alon U. (2015). Evolution of bow-tie architectures in biology. PLOS Comput. Biol. 11 (3), e1004055. 10.1371/journal.pcbi.1004055 25798588 PMC4370773

[B28] Futuyma D. J. Kirkpatrick M. (2022). Evolution. 5th edn. New York: Oxford University Press.

[B29] Gorbunova V. Seluanov A. Mita P. McKerrow W. Fenyö D. Boeke J. D. (2021). The role of retrotransposable elements in ageing and age-associated diseases. Nature 596 (7870), 43–53. 10.1038/s41586-021-03542-y 34349292 PMC8600649

[B30] Gould S. J. (2002). The Structure of Evolutionary Theory. Cambridge, MA: Harvard University Press.

[B31] Hackett J. A. Surani M. A. (2013). Beyond DNA: programming and inheritance of parental methylomes. Cell 153 (4), 737–739. 10.1016/j.cell.2013.04.044 23663772 PMC4338575

[B32] Hanahan D. (2022). Hallmarks of cancer: new dimensions. Cancer Discov. 12 (1), 31–46. 10.1158/2159-8290.CD-21-1059 35022204

[B33] Hanahan D. Weinberg R. A. (2011). Hallmarks of cancer: the next generation. Cell 144 (5), 646–674. 10.1016/j.cell.2011.02.013 21376230

[B34] Hansen T. F. Houle D. (2004). “Evolvability, stabilizing selection, and the problem of stasis,” in Phenotypic Integration: Studying the Ecology and Evolution of Complex Phenotypes. Editors Pigliucci M. Preston K. (New York: Oxford University Press), 130–150. 10.1093/oso/9780195160437.003.0006

[B35] Heng J. Heng H. H. (2022). Genome chaos: creating new genomic information essential for cancer macroevolution. Seminars Cancer Biol. 81, 160–175. 10.1016/j.semcancer.2020.11.003 33189848

[B36] Heng J. Heng H. H. (2023). Karyotype as code of codes: an inheritance platform to shape the pattern and scale of evolution. Biosystems 233, 105016. 10.1016/j.biosystems.2023.105016 37659678

[B37] Henikoff S. Ahmad K. Malik H. S. (2001). The centromere paradox: stable inheritance with rapidly evolving DNA. Science 293 (5532), 1098–1102. 10.1126/science.1062939 11498581

[B38] Holohan C. Van Schaeybroeck S. Longley D. B. Johnston P. G. (2013). Cancer drug resistance: an evolving paradigm. Nat. Rev. Cancer 13 (10), 714–726. 10.1038/nrc3599 24060863

[B39] Hunt G. (2007). The relative importance of directional change, random walks, and stasis in the evolution of fossil lineages. Proc. Natl. Acad. Sci. 104 (47), 18404–18408. 10.1073/pnas.0704088104 18003931 PMC2141789

[B40] Ishina I. A. Zakharova M. Y. Kurbatskaia I. N. Mamedov A. E. Belogurov A. A. Jr. Gabibov A. G. (2023). MHC class II presentation in autoimmunity. Cells 12 (2), 314. 10.3390/cells12020314 36672249 PMC9856717

[B41] Jablonka E. (2002). Information: its interpretation, its inheritance, and its sharing. Philosophy Sci. 69, 578–605. 10.1086/344621

[B42] Kelleher F. C. Fennelly D. Rafferty M. (2006). Common critical pathways in embryogenesis and cancer. Acta Oncol. 45 (4), 375–388. 10.1080/02841860600602946 16760173

[B43] Kitano H. Oda K. (2006). Robustness trade-offs and host–microbial symbiosis in the immune system. Mol. Syst. Biol. 2, 0022. 10.1038/msb4100039 16738567 PMC1681473

[B44] Kmita M. Duboule D. (2003). Organizing axes in time and space; 25 years of colinear tinkering. Science 301 (5631), 331–333. 10.1126/science.1085753 12869751

[B45] Lakatos I. (1970). “Falsification and the methodology of scientific research programmes,” in Criticism and the Growth of Knowledge. Editors Lakatos I. Musgrave A. (Cambridge: Cambridge University Press), 91–196.

[B46] Laland K. N. Uller T. Feldman M. W. Sterelny K. Müller G. B. Moczek A. (2015). The extended evolutionary synthesis: its structure, assumptions and predictions. Proc. R. Soc. B 282, 20151019. 10.1098/rspb.2015.1019 26246559 PMC4632619

[B47] Laouris Y. (2026). Membranes as the earliest entropy-resisting structures in the origin of life. Discov. Life 56, 17. 10.1007/s11084-026-09730-y

[B48] Lipton P. (2004). Inference to the Best Explanation. 2nd edn. London: Routledge.

[B49] López-Otín C. Blasco M. A. Partridge L. Serrano M. Kroemer G. (2013). The hallmarks of aging. Cell 153 (6), 1194–1217. 10.1016/j.cell.2013.05.039 23746838 PMC3836174

[B50] Malik H. S. Henikoff S. (2009). Major evolutionary transitions in centromere complexity. Cell 138 (6), 1067–1082. 10.1016/j.cell.2009.08.036 19766562

[B51] Malone C. D. Hannon G. J. (2009). Small RNAs as guardians of the genome. Cell 136 (4), 656–668. 10.1016/j.cell.2009.01.045 19239887 PMC2792755

[B52] Mao H. Zhao X. Sun S.-C. (2025). NF-κB in inflammation and cancer. Cell. and Mol. Immunol. 22 (8), 811–839. 10.1038/s41423-025-01310-w 40562870 PMC12310982

[B53] McKinley K. L. Cheeseman I. M. (2016). The molecular basis for centromere identity and function. Nat. Rev. Mol. Cell Biol. 17 (1), 16–29. 10.1038/nrm.2015.5 26601620 PMC8603311

[B54] Medzhitov R. (2008). Origin and physiological roles of inflammation. Nature 454, 428–435. 10.1038/nature07201 18650913

[B55] Menche J. Sharma A. Kitsak M. Ghiassian S. D. Vidal M. Loscalzo J. (2015). Uncovering disease-disease relationships through the incomplete interactome. Science 347 (6224), 1257601. 10.1126/science.1257601 25700523 PMC4435741

[B56] Milo R. Shen-Orr S. Itzkovitz S. Kashtan N. Chklovskii D. Alon U. (2002). Network motifs: simple building blocks of complex networks. Science 298 (5594), 824–827. 10.1126/science.298.5594.824 12399590

[B57] Moreno A. Mossio M. (2015). Biological Autonomy: A Philosophical and Theoretical Enquiry. Dordrecht: Springer.

[B58] Müller G. B. (2017). Why an extended evolutionary synthesis is necessary. Interface Focus 7 (5), 20170015. 10.1098/rsfs.2017.0015 28839929 PMC5566817

[B59] Oda K. Kitano H. (2006). A comprehensive map of the toll-like receptor signaling network. Mol. Syst. Biol. 2, 0015. 10.1038/msb4100057 16738560 PMC1681489

[B60] Ohno S. (1972). So much ‘junk’ DNA in our genome. Brookhaven Symposia Biol. 23, 366–370. 5065367

[B61] Orgel L. E. Crick F. H. C. (1980). Selfish DNA: the ultimate parasite. Nature 284, 604–607. 10.1038/284604a0 7366731

[B62] Ozata D. M. Gainetdinov I. Zoch A. O’Carroll D. Zamore P. D. (2019). PIWI-Interacting RNAs: small RNAs with big functions. Nat. Rev. Genet. 20 (2), 89–108. 10.1038/s41576-018-0073-3 30446728

[B63] Pakos-Zebrucka K. Koryga I. Mnich K. Ljujic M. Samali A. Gorman A. M. (2016). The integrated stress response. EMBO Rep. 17 (10), 1374–1395. 10.15252/embr.201642195 27629041 PMC5048378

[B64] Patke A. Young M. W. Axelrod S. (2020). Molecular mechanisms and physiological importance of circadian rhythms. Nat. Rev. Mol. Cell Biol. 21 (2), 67–84. 10.1038/s41580-019-0179-2 31768006

[B65] Pavlova N. N. Zhu J. Thompson C. B. (2022). The hallmarks of cancer metabolism: still emerging. Cell Metab. 34 (3), 355–377. 10.1016/j.cmet.2022.01.007 35123658 PMC8891094

[B66] Pearson J. C. Lemons D. McGinnis W. (2005). Modulating hox gene functions during animal body patterning. Nat. Rev. Genet. 6 (12), 893–904. 10.1038/nrg1726 16341070

[B67] Peirce C. S. (1931). “Collected papers of charles sanders peirce, vols. 1–8,”. Cambridge, MA: Harvard University Press.

[B68] Peter I. S. Davidson E. H. (2011). Evolution of gene regulatory networks controlling body plan development. Cell 144 (6), 970–985. 10.1016/j.cell.2011.02.017 21414487 PMC3076009

[B69] Peter I. S. Davidson E. H. (2017). Assessing regulatory information in developmental gene regulatory networks. Proc. Natl. Acad. Sci. U. S. A. 114, 5862–5869. 10.1073/pnas.1610616114 28584110 PMC5468647

[B70] Pievani T. (2012). “An evolving research programme: the structure of evolutionary theory from a Lakatosian perspective,” in The Theory of Evolution and its Impact. Editor Fasolo A. (Milano: Springer), 211–228. 10.1007/978-88-470-1974-4_14

[B71] Pigliucci. M. Müller G. B. (2010). Evolution: The Extended Synthesis (Cambridge, MA: MIT Press).

[B72] Plato (1997). “Timaeus,” in Plato: Complete Works, Editor. J. M. Cooper, trans. D. J. Zeyl. Indianapolis: Hackett Publishing.

[B73] Reik W. Walter J. (2001). Genomic imprinting: parental influence on the genome. Nat. Rev. Genet. 2 (1), 21–32. 10.1038/35047554 11253064

[B74] Sedlazeck F. J. Lee H. Darby C. A. Schatz M. C. (2018). Piercing the dark matter: bioinformatics of long-range sequencing and mapping. Nat. Rev. Genet. 19 (6), 329–346. 10.1038/s41576-018-0003-4 29599501

[B75] Seo H.-C. Edvardsen R. B. Maeland A. D. Bjordal M. Jensen M. F. Hansen A. (2004). Hox cluster disintegration with persistent anteroposterior order of expression in *Oikopleura dioica* . Nature 431, 67–71. 10.1038/nature02709 15343333

[B76] Shapiro J. A. (2011). Evolution: A View from the 21st Century. Upper Saddle River, NJ: FT Press.

[B77] Shubin N. Tabin C. Carroll S. (2009). Deep homology and the origins of evolutionary novelty. Nature 457 (7231), 818–823. 10.1038/nature07891 19212399

[B78] Simpson G. G. (1944). Tempo and Mode in Evolution. New York: Columbia University Press.

[B79] Takahashi J. S. (2017). Transcriptional architecture of the Mammalian circadian clock. Nat. Rev. Genet. 18 (3), 164–179. 10.1038/nrg.2016.150 27990019 PMC5501165

[B80] Tanaka R. Csete M. Doyle J. (2005). Highly optimised global organisation of metabolic networks. IEE Proc. — Syst. Biol. 152 (4), 179–184. 10.1049/ip-syb:20050042 16986258

[B81] Tieri P. Grignolio A. Zaikin A. Mishto M. Remondini D. Castellani G. C. (2010). Network, degeneracy and bow tie. Integrating paradigms and architectures to grasp the complexity of the immune system. Theor. Biol. Med. Model. 7, 32. 10.1186/1742-4682-7-32 20701759 PMC2927512

[B82] Waddington C. H. (1957). The Strategy of the Genes. London: Allen and Unwin.

[B83] Wagner A. (2005). Robustness and Evolvability in Living Systems. Princeton, NJ: Princeton University Press.

[B84] Weinberg R. A. (2023). The Biology of Cancer. 3rd edn. New York: W. W. Norton and Company.

[B85] Weiss M. C. Sousa F. L. Mrnjavac N. Neukirchen S. Roettger M. Nelson-Sathi S. (2016). The physiology and habitat of the last universal common ancestor. Nat. Microbiol. 1, 16116. 10.1038/nmicrobiol.2016.116 27562259

[B86] Whewell W. (1840). The Philosophy of the Inductive Sciences, Founded upon Their History. London: John W. Parker.

[B87] Wimsatt W. C. (1986). “Developmental constraints, generative entrenchment, and the innate-acquired distinction,” in Integrating Scientific Disciplines. Editor Bechtel W. (Dordrecht: Martinus Nijhoff), 185–208.

[B88] Xavier J. C. Gerhards R. E. Wimmer J. L. E. Brueckner J. Tria F. D. K. Martin W. F. (2021). The metabolic network of the last bacterial common ancestor. Commun. Biol. 4, 413. 10.1038/s42003-021-01918-4 33772086 PMC7997952

[B89] Zoghbi H. Y. Beaudet A. L. (2016). Epigenetics and human disease. Cold Spring Harb. Perspect. Biol. 8 (2), a019497. 10.1101/cshperspect.a019497 26834142 PMC4743078

